# Traditional versus blended CPR training program: A randomized controlled non-inferiority study

**DOI:** 10.1038/s41598-020-67193-1

**Published:** 2020-06-22

**Authors:** Cheng-Yu Chien, Shao-Yu Fang, Li-Heng Tsai, Shang-Li Tsai, Chen-Bin Chen, Chen-June Seak, Yi-Ming Weng, Chi-Chun Lin, Wei-Che Chien, Chien-Hsiung Huang, Cheng-Yu Lin, Chung-Hsien Chaou, Peng-Huei Liu, Hsiao-Jung Tseng, Jih-Chang Chen, Shu-Yuan Peng, Tsung-Hsuan Cheng, Kuang-Hung Hsu, Chip-Jin Ng

**Affiliations:** 1 Department of Emergency Medicine, Chang Gung Memorial Hospital, Linkou and College of Medicine, Chang Gung University, Taoyuan, 333 Taiwan; 2 Department of Emergency Medicine, Ton-Yen General Hospital, Zhubei, 302 Taiwan; 3 Department of Emergency Medicine, Chang Gung Memorial Hospital Taipei Branch, Taipei, 105 Taiwan; 4 Department of Emergency Medicine, Taoyuan General Hospital, Ministry of Health and Welfare, Taoyuan, 330 Taiwan; 5 Biostatistics Unit, Clinical Trial Center, Chang Gung Memorial Hospital, Linkou, Taoyuan, 333 Taiwan; 6 Department of nursing, Ton-Yen General Hospital, Zhubei, 302 Taiwan; 7 Laboratory for Epidemiology, Chang Gung University, Taoyuan, 333 Taiwan; 8 Department of Urology, Chang Gung Memorial Hospital, Linkou and College of Medicine, Chang Gung University, Taoyuan, 333 Taiwan

**Keywords:** Outcomes research, Randomized controlled trials

## Abstract

Cardiopulmonary resuscitation (CPR) training and its quality are critical in improving the survival rate of cardiac arrest. This randomized controlled study investigated the efficacy of a newly developed CPR training program for the public in a Taiwanese setting. A total of 832 adults were randomized to either a traditional or blended (18-minute e-learning plus 30-minute hands-on) compression-only CPR training program. The primary outcome was compression depth. Secondary outcomes included CPR knowledge test, practical test, quality of CPR performance, and skill retention. The mean compression depth was 5.21 cm and 5.24 cm in the blended and traditional groups, respectively. The mean difference in compression depth between groups was −0.04 (95% confidence interval −0.13 to infinity), demonstrating that the blended CPR training program was non-inferior to the traditional CPR training program in compression depth after initial training. Secondary outcome results were comparable between groups. Although the mean compression depth and rate were guideline-compliant, only half of the compressions were delivered with adequate depth and rate in both groups. CPR knowledge and skill retained similarly in both groups at 6 and 12 months after training. The blended CPR training program was non-inferior to the traditional CPR training program. However, there is still room for improvement in optimizing initial skill performance as well as skill retention. Clinical Trial Registration: NCT03586752; www.clinicaltrial.gov

## Introduction

The survival rate of out-of-hospital cardiac arrest (OHCA) is low. In the United States, it has remained between 7% and 9% for the past decades^1^. Meanwhile the 180-day OHCA survival rate was reported to be 9.8% in Taiwan^2^.

Early defibrillation is a treatment option that can increase OHCA survival rate and survival outcomes^3^. Ever since its promotion by the American Heart Association (AHA)^4^, many countries have installed automated external defibrillators (AEDs) in public or private places including tourists spots, shopping malls, airports, casinos, schools, offices and so forth, with increased coverage and accessibility. In Taiwan, up until 2017, a total of 8334 AEDs had been installed nationwide^5^. Wang *et al*.^5^ reported that, among the documented OHCA cases with AEDs used, 35% were known to be operated by the employees at the designated AED locations, and long-term care facilities had the highest utilization rate of AED. In addition, high-quality chest compressions during cardiopulmonary resuscitation (CPR) also improve OHCA patient outcomes^6–8^. However, studies have shown the quality of CPR to be substandard^9,10^. Therefore, training with a focus on cardiopulmonary resuscitation (CPR) quality and AED should be implemented and provided, particularly at the AED locations of high cardiac arrest frequency.

Traditionally, the CPR-AED training course is instructor-led and classroom-based. For both the trainer and course-taker, time, cost, logistics, and discomfort over being in a classroom setting are often barriers to the traditional training program^11^. With technological advancements, new training formats are evolving. Numerous studies have been conducted to identify improvements in training formats as well as the quality of CPR skills in different populations^11–13^. However, there is still no consensus about how to train the public and the optimal frequency of CPR-AED training.

In the present study, we aimed to develop an alternative training format, which was time-saving and convenient, while maintaining high quality, for the Taiwanese population. By adopting a non-inferiority study design, we hypothesized that the new CPR-AED training format would be comparable to that of the traditional program in terms of CPR quality (e.g. compression depth) as well as CPR knowledge and other skills performance but would be more time-saving and cost-effective.

## Results

### Study population

**Figure 1:**
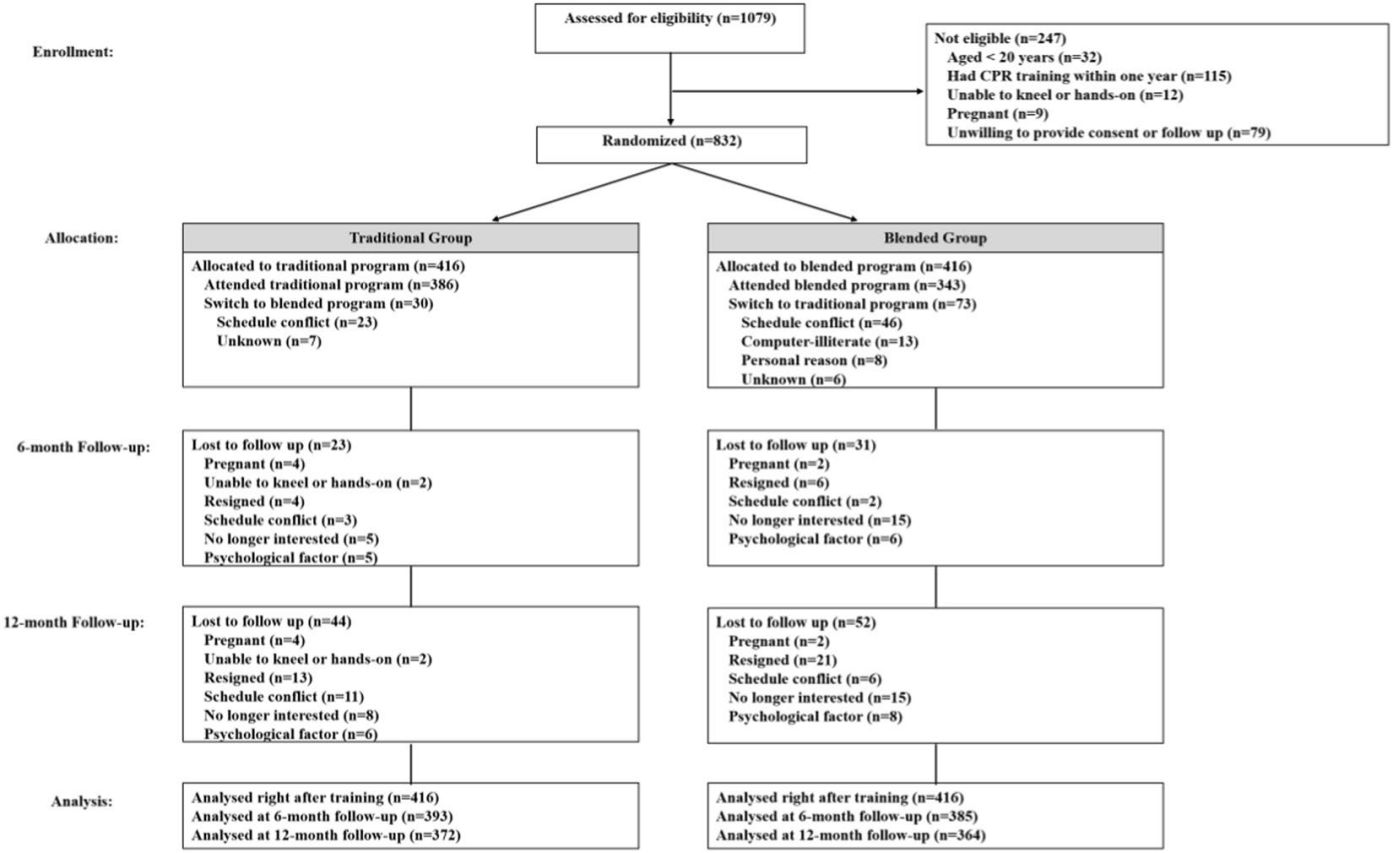
Study flow diagram.

The participant flow chart according to the 2017 CONSORT statement^15^ is displayed in Fig. 1. A total of 1079 subjects were screened for eligibility, 832 of whom were enrolled and randomly assigned to either the traditional or blended program for CPR training. Of the 416 participants assigned to either of the groups, 372 in the traditional group and 364 in the blended group completed the 12-month follow-up reassessment. As summarized in Table 1, the demographic characteristics of participants in the traditional and blended groups were comparable and did not differ significantly.

**Table 1:** Characteristics of participants.

Characteristics	Traditional Group (n = 416)	Blended Group (n = 416)	*p*-value
Age, years	37.40 (10.78)	37.27 (10.22)	0.88
Height, cm	160.63 (6.68)	160.77 (7.36)	0.80
Weight, kg	61.06 (15.61)	60.75 (11.43)	0.77
Sex, n (%)			0.82
Female	291 (70.0%)	288 (69.2%)	
Male	125 (30.0%)	128 (30.8%)	
Education level, n (%)			0.57
Less than high school	20 (4.8%)	15 (3.6%)	
High school	127 (30.5%)	125 (30.0%)	
College	269 (64.7%)	276 (66.3%)	
Marital status, n (%)			0.65
Single	168 (40.4%)	157 (37.7%)	
Married	228 (54.8%)	236 (56.7%)	
Divorced	12 (2.9%)	17 (4.1%)	
Widowed	8 (1.9%)	6 (1.4%)	
Religion, n (%)			0.77
No	231 (55.5%)	248 (59.6%)	
Buddhism	99 (23.8%)	90 (21.6%)	
Christian	21 (5.0%)	19 (4.6%)	
Other	65 (15.6%)	59 (14.2%)	
Exercise habits, n (%)	113 (27.2%)	123 (29.6%)	0.44
Last CPR training, years	1.80 (0.93)	1.92 (1.05)	0.12
Written test total score, %	70.99 (9.7)	70.88 (9.48)	0.87
CPR section, %	63.18 (12.01)	62.22 (14.53)	0.30
AED section, %	73.72 (20.48)	72.76 (21.23)	0.51
Others, %	74.42 (16.15)	73.75 (15.83)	0.55

Data are expressed as mean (SD) or n (%). Abbreviations: cm, centimeter; kg, kilogram; n, number; CPR, cardiopulmonary resuscitation; AED, automated external defibrillator; SD, standard deviation.

### Right after training

Regarding the primary outcome, the mean (SD) compression depth was 5.21 (0.66) cm in the blended group and 5.24 (0.63) cm in the traditional group (Table 2). With the predefined margin of 0.18 cm, the mean difference in compression depth between groups was −0.04 cm (95% confidence interval −0.13 to infinity, p = 0.006), supporting the alternative hypothesis that the blended CPR training program was non-inferior to the traditional program.

**Table 2:** CPR performance after training.

	Traditional Group (n = 416)	Blended Group (n = 416)	Mean difference [95% CI]	*p*-value
Primary outcome				
Compression depth, cm	5.24 (0.63)	5.21 (0.66)	−0.04 [−0.13, inf]	0.006*
Secondary outcome				
Written test total score, %	89.22 (9.12)	88.35 (10.03)	−0.88 [−2.35, 0.59]	0.19
CPR section, %	91.75 (12.00)	90.78 (12.33)	−0.97 [−1.85, 3.79]	0.25
AED section, %	88.92 (27.67)	87.19 (26.78)	−1.73 [−6.77, 5.92]	0.36
Others, %	90.91 (10.76)	91.18 (12.47)	0.27 [−2.45, 3.31]	0.74
Practical test score	34.44 (3.46)	34.88 (2.23)	0.44 [−0.01, 0.88]	0.54
Compression rate, bpm	113.17 (13.03)	114.56 (11.37)	1.39 [−0.63, 3.40]	0.18
Full chest recoil, %	75.7 (32.0)	77.2 (31.0)	1.59 [−3.59, 6.78]	0.33
Adequate depth, %	55.8 (34.6)	54.5 (36.5)	−1.36 [−7.22, 4.06]	0.65
Adequate rate, %	56.4 (33.2)	54.3 (32.9)	−2.01 [−7.45, 3.44]	0.47

Data are expressed as mean (SD) unless otherwise specified. *Only the primary outcome was tested by using the one-sided non-inferior test. Abbreviations: CPR, cardiopulmonary resuscitation; AED, automated external defibrillator; bpm, beats per minute; cm, centimeter; n, number; SD, standard deviation; inf, infinity.

**Figure 2:**
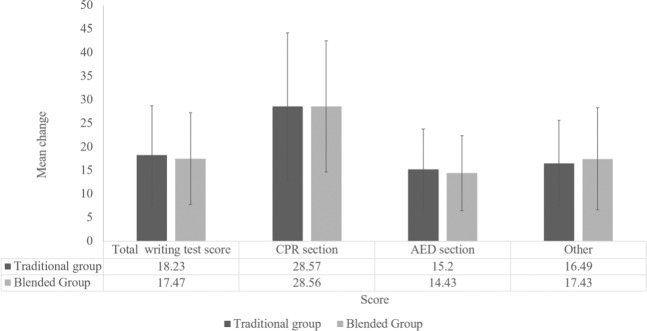
Mean change in written test score from baseline at right after training. CPR, cardiopulmonary resuscitation; AED, automated external defibrillation.

Regarding other outcomes, the mean (SD) written test score was 88.35 (10.03), practical test score was 34.88 (2.23), compression rate was 114.56 (11.37) bpm, full chest recoil was 77.2%, and mean percentage of compressions delivered with adequate depth and rate was 54% in the blended group. These results of CPR performance in the blended group were comparable to those in the traditional group with no significant difference (Table 2). The mean change in written test score from baseline to right after training between groups were comparable as depicted in Fig. 2. The proportion of participants who carried out each BLS sequence in both the traditional and blended group did not differ significantly either (Table 3).

**Table 3:** CPR and AED skill testing after training.

	Traditional Group (n = 416)	Blended Group (n = 416)	*p*-value
Confirm scene safety	389 (93.5%)	399 (95.9%)	0.12
Check consciousness	398 (95.9%)	393 (94.7%)	0.42
Call for help and AED	392 (94.2%)	397 (95.4%)	0.43
Check breathing	348 (83.7%)	338 (81.3%)	0.36
CPR location	401 (96.4%)	402 (96.6%)	0.85
CPR posture	401 (96.4%)	401 (96.4%)	>0.99
AED operating	377 (90.6%)	374 (89.9%)	0.73
AED pad location	400 (96.2%)	392 (94.2%)	0.20

Data are expressed as n (%). Abbreviations: CPR, cardiopulmonary resuscitation; AED, automated external defibrillator; n, number; SD, standard deviation.

### Follow-up

There was no significant difference in the retention of CPR skills between groups, as shown in Table 4. The results of CPR performance of the groups at 6-month and 12-month follow-up testing were comparable.

**Table 4:** CPR performance comparisons by group at 6-month and 12-month follow-up.

	Follow-up	Traditional Group	Blended Group	*p*-value
Written test score	6-month	80.80 (12.06)	80.29 (13.09)	0.80
	12-month	79.84 (12.17)	78.36 (13.56)	0.50
Practical test score	6-month	29.96 (4.97)	30.01 (4.59)	0.95
	12-month	27.93 (3.45)	28.36 (5.89)	0.82
Compression depth, cm	6-month	5.09 (0.83)	5.00 (0.67)	0.45
	12-month	5.22 (0.81)	4.99 (0.79)	0.12
Compression rate, bpm	6-month	124.28 (17.76)	124.14 (16.05)	0.96
	12-month	120.68 (13.22)	124.94 (15.28)	0.07
Full chest recoil, %	6-month	63.5 (39.9)	64.6 (39.3)	0.86
	12-month	70.1 (34.6)	71.0 (35.4)	0.89
Adequate depth, %	6-month	52.7 (37.2)	47.1 (36.8)	0.36
	12-month	47.3 (40.3)	43.4 (35.7)	0.58
Adequate rate, %	6-month	28.5 (32.7)	31.7 (35.4)	0.58
	12-month	40.7 (34.1)	30.9 (34.7)	0.08

Data are expressed as mean (SD). The number of participants analyzed at 6-month and 12-month follow-up were 393 and 372, respectively, in the traditional group, while 385 and 364, respectively, in the blended group. Abbreviations: bpm, beats per minute; cm, centimeter; n, number; SD, standard deviation.

At six months after training, the mean (SD) written test score of the traditional and blended groups dropped to 80.80 (12.06) and 80.29 (13.09), respectively (p = 0.80). Meanwhile, the mean (SD) practical test score of the traditional and blended group dropped to 29.96 (4.97) and 30.01 (4.59), respectively (p = 0.50). Participants in the traditional and blended groups performed CPR with a mean (SD) compression depth of 5.09 (0.83) cm and 5.00 (0.67) cm, compression rate of 124.28 (17.76) bpm and 124.14 (16.05) bpm, and full chest recoil of 63.5% and 64.6%, respectively. The mean percentage of compressions delivered with adequate depth was 52.7% in the traditional group and 47.1% in the blended group (p = 0.36). The mean percentage of compressions delivered with adequate rate was 28.5% in the traditional group and 31.7% in the blended group (p = 0.58).

At 12 months after training, the mean (SD) written test scores of the traditional and blended groups were 79.84 (12.17) and 78.36 (13.56), respectively (p = 0.50). Meanwhile, the mean (SD) practical test scores of the traditional and blended group were 27.93 (3.45) and 28.36 (5.89), respectively (p = 0.82). The mean (SD) compression depth was 5.22 (0.81) cm and 4.99 (0.79) cm, compression rate was 120.68 (13.22) bpm and 124.94 (15.28) bpm, full chest recoil was 70.1% and 71.0%, percentage of compressions delivered with adequate depth was 47.3% and 43.4%, and the percentage of compressions delivered with adequate rate was 40.7% and 30.9% in the traditional group and blended group, respectively.

## Discussion

We designed a blended CPR training program and investigated its effect on the participants’ CPR knowledge and skills performance in comparison to the traditional CPR training program. The results demonstrated non-inferiority for the primary outcome of mean compression depth. The theoretical knowledge, practical skills, and the quality of CPR were comparable in both groups throughout the study duration, indicating that the blended CPR training program is as effective as the traditional CPR training program.

Our results showed that a concise theory module yielded the same effect on the participants’ CPR knowledge as the traditional program, though with less time spent (18 minutes vs 60 minutes) and less effort. The retention of CPR knowledge was similar at 6 and 12 months after training in both groups. The participants’ CPR knowledge slightly reduced at the 6-month follow-up and then was retained until 12 months after training. Overall, the participants’ CPR knowledge remained at a sufficient level.

Previous studies have shown that participants in a training program without hands-on practice demonstrate poorer CPR skills performance^16,17^. Einspruch *et al*.^11^ demonstrated that just after a brief practice-while-watching self-training, participants can perform CPR with adequate skill. Given that practical training and hands-on time are essential in CPR training to optimize CPR skills performance, our blended program also comprised a 30-minute hands-on practice session. The length of hands-on time was equivalent to the traditional program.

Our findings on CPR skills showed that the CPR performed by participants in the blended program was of similar quality to those in the traditional program. Participants in both groups performed CPR skill at an acceptable level after initial training, i.e., had a mean compression depth that was between 5 cm and 6 cm and a mean compression rate that was between 100 bpm and 120 bpm. Of all the compressions performed, approximately 75% allowed full chest recoil and 55% with adequate compression depth and rate, which is comparable to the finding of Fernando *et al*.^9^.

Consistent with previous studies^18–21^, our study also found that CPR skills deteriorate faster than theoretical knowledge. During the follow-up period, the mean compression depth did not meet the lower limit of 5 cm in the blended group. In both groups, the mean compression rate exceeded the upper limit of the guideline-recommended range of 100–120 bpm, the percentage of full chest recoil reduced by 6–13%, the percentage of compression performed with adequate depth reduced by 3–11%, and the percentage of compression performed with adequate rate reduced by 15–27%.

As such, a retraining or refreshing course should be offered more frequently and at shorter intervals to retain CPR skills. Woollard *et al*.^22^ showed that skill retention was higher in the group taking a second refresher class at 7 months compared with 12 months and recommended an interval for refresher training of no longer than 7 months. Nishiyama *et al*.^23^ revealed that a 15-minute refresher training provided at six months after initial training can retain participants’ CPR skills for up to one year. Anderson *et al*.^24^ reported that the shorter the interval of refresher training, the higher the proportion of participants able to perform high-quality CPR. As the target learners of our CPR training program are laypersons whose workplaces are located near an AED, minimal disruption of the working day, workplace support, and cost for training are critical factors that should be taken into account when refresher training is offered. Based on the above-mentioned findings and ours, a refresher training of 30-minute hands-on practice only delivered at a 6-month interval would be an optimal training length and frequency for CPR skill retention for our target learners.

In our study, about half of the compressions were performed with adequate depth and rate after initial training, implying room for improvement in immediate skill performance. To boost CPR skills in the initial training, it is suggested that a hands-on practice that fosters the achievement of excellent CPR—greater than or equal to 90% of compressions are guideline-compliant—can be implemented^24^. Moreover, training focus on compression rate can be emphasized^25^, given that it was the most deteriorated metric among the three high-quality CPR metrics.

This study has several limitations. The study participants were recruited from the mandated areas of AED installation declared by the Ministry of Health and Welfare of Taiwan and where AEDs were donated by the Chang Gung Memorial Hospital; thus, selection bias may have been introduced. The study used intention-to-treat analysis; 12% (103/832) of participants attended a training program that they were not initially assigned to. The participants’ mean body weight was around 61 kg in our study, thus it was not known whether participants with low body weight could achieve high-quality CPR performance or not. Although there was no upper limit of age in subject recruitment, all participants in this study were below 50 years of age, hence whether this blended CPR training program would be suitable for older adults or elderly is yet to be determined. Lastly, the participants were informed of the day of reassessment for follow-up and did not need to revise to assess their retention of CPR knowledge and skill. However, it was not known whether the participants revised or practiced before the reassessment.

In conclusion, the blended CPR training program was non-inferior to the traditional CPR training program in terms of participants’ CPR compression depth, while the other CPR knowledge and skills were comparable. This briefer and less labor-intensive blended CPR program can be used as an alternative training option and could be used as a refresher training at an optimal interval. However, there is still room for improvement in optimizing initial CPR skill performance as well as skill retention.

## Methods

### Study design, setting, participants

This was a randomized, controlled, single-blinded study. Enrollment was conducted from June 2016 to November 2017. The study was completed in 2018. Participants were recruited from the 250 recipients of AED donated by Chang Gung Memorial Hospital between the years 2012 and 2014 in Taiwan. To be included in the study, the participants had to: a) be aged at least 20 years and b) not have attended any CPR training for at least a year prior to enrollment. Participants were excluded if they were unable to kneel to perform CPR, were pregnant, or not willing to sign an informed consent form. Written informed consent was obtained from all participants before any study-related procedure was carried out. The study was approved by the Chang Gung Memorial Foundation Institutional Review Board (approval number: 201600149B0 and 201900399B0) and performed in accordance with all relevant guidelines and regulatory requirements. This study was registered on ClinicalTrials.gov (NCT03586752).

### Randomization and blinding

After obtaining informed consent and confirming eligibility, participants were randomly assigned following simple randomization procedures. A computer-generated randomization list was used for the allocation of the participants. Both the participants and instructors were aware of the assignment to the training program, while the examiners were kept blinded to it.

### Interventions

The traditional program is an instructor-led classroom-based training using the practice-while-learning format. It takes 90 minutes in total; a 60-minute CPR knowledge lesson which include the the CPR lecture and demo, AED and demo, introduction of the law, and a 30-minute hands-on session for compression-only CPR.

The blended program was developed by the Taiwan Society of Emergency Medicine and approved by the chairman of Taiwan Society of Emergency Medicine. It consisted of a combination of an 18-minute e-learning module and a 30-minute hands-on session for compression-only CPR. The 18-minute e-learning module was a video introducing the cardiac arrest scene, how and why to hand-only CPR, the benefit of CPR and AED in OHCA, CPR and AED steps, and introduction of the law, which were the core knowledge of CPR and AED. A link to access the e-learning video was provided to the participants assigned to the blended program three days before the hands-on session took place. The participants had to log in to view the course material and pass the e-learning assessment at the end of the e-learning module. Upon completion of the e-learning module, participants had to complete an instructor-led hands-on session for 30 minutes in class.

The instructors teaching in both CPR training programs were an emergency physician as well as an AHA instructor. The main difference between the blended program and the traditional program was that the time and format of the CPR and AED knowledge lecture. The CPR and AED knowledge lecture in the blended program was concentrated to 18 minutes to include only the core knowledge and delivered in a video format. Participants in the blended program could learn the CPR knowledge in a lesser time (18 minutes vs 60 minutes in the traditional program) at anywhere as long as there was an internet access (classroom based in the traditional program). The time and format for the hands-on session was the same in both the traditional and blended programs (30 minutes in class).

Sensor-equipped manikin (Resusci Anne with QCPR, Laerdal Medical AS, Norway) was used in hands-on practice in both groups. The ratio of participants to manikins to instructors per class was 8:4:1. There were four instructors and six examiners in this study. No instructor served as an examiner or vice versa.

### Outcome measures

The study assessed the participants’ knowledge and performance of CPR following training and the retaining of CPR knowledge and skills at 6 and 12 months after training.

CPR knowledge was assessed by a written test. The written test consisted of 15 multiple choice questions with a total score of 100. CPR performance was assessed in two ways: examiner-rated and manikin feedback. Examiners assessed the participants’ performance in terms of the adequacy of administering BLS sequence from verifying scene safety to AED use one by one. The maximum score of the examiner-rated practical test was 40. Objective data on the quality of CPR including compression depth, compression rate, and full chest recoil were recorded from the manikin feedback. Pursuant to 2015 AHA guidelines update for CPR and emergency cardiovascular care (ECC)^14^, a high-quality CPR is defined as: (1) compression depth of 5–6 cm, (2) compression rate of 100–120 beats per minute (bpm), and (3) allow full chest wall recoil. As compression-only CPR is recommended, ventilation was not performed and thus was not assessed in this study.

The primary outcome measure was the difference in compression depth between groups to demonstrate non-inferiority. The secondary outcome measures were the written test score, examiner-rated practical test score, compression depth, compression rate, full chest recoil, the percentage of compressions delivered with adequate depth (5–6 cm), percentage of compressions delivered with adequate rate (100–120 bpm), and the BLS skill testing.

### Sample size

The study hypothesized that the compression depth performed by participants attending the blended program would not be inferior to the compression depth performed by those attending the traditional program. Assuming a significance level of 0.05 and a non-inferiority margin of 0.18 (based on our non-published pilot study), 328 participants per arm were required to achieve a statistical power of 95%. Considering a dropout rate of 20%, 410 participants per arm, 820 participants in total, were planned to be enrolled.

### Statistical methods

The study used intention-to-treat analysis which involved all participants randomly assigned to either the traditional or blended programs. Categorical variables were presented as numbers and percentages and were compared using a Chi-squared test or Fisher’s exact test, as appropriate. Continuous variables were presented as means and standard deviations and were compared using two sample independent t-test. The non-inferiority margin for the mean difference in the compression depth was defined at 0.18 cm after initial training. The null hypothesis would be the mean difference in the compression depth between the blended program and traditional program exceeded 0.18 cm. Except for testing compression depth with the one-sided non-inferior test, the others were two-sided tests. Significance level α was set at 0.05. The data were analyzed using IBM SPSS Statistics (version 25.0 for Windows; IBM Corp., Armonk, NY USA).

## Supplementary information

Supplementary Information.

Supplementary Information.
