# Supplementary material for: Traditional versus blended CPR training program: A randomized controlled non-inferiority study
Source: Sci Rep. 2020 Jun 22;10:10032. doi: 10.1038/s41598-020-67193-1 (PMC7308401; doi:10.1038/s41598-020-67193-1)
Supplement: Supplementary file 1 — Supplementary Information. [file 41598_2020_67193_MOESM1_ESM.pdf]

**CPR Practice (Skill Testing) Checklist**

Student Name: ______________________________

Affiliation: _________________________________

Date of Test： ___________________(MM/DD/YYYY)

Native Language:  Mandarin  Taiwanese  Hakka  Other_______

Course Subject Evaluation Form (to be filled by the examiner)

<table>
<tr><th>Step</th><th>Item</th><th colspan="2">YES</th><th colspan="2">NO</th></tr>
<tr><td>1</td><td>Verbally confirm that the scene is safe</td><td colspan="2">2</td><td colspan="2">0</td></tr>
<tr><td>2</td><td colspan="5">Checks responsiveness</td></tr>
<tr><td></td><td>Confirm response: tap the patient</td><td colspan="2">2</td><td colspan="2">0</td></tr>
<tr><td></td><td>Confirm response: call the patient’s name loudly</td><td colspan="2">2</td><td colspan="2">0</td></tr>
<tr><td>3</td><td colspan="5">Calls for help</td></tr>
<tr><td></td><td>Ask someone to call 911</td><td colspan="2">2</td><td colspan="2">0</td></tr>
<tr><td></td><td>Ask someone to get an AED</td><td colspan="2">2</td><td colspan="2">0</td></tr>
<tr><td>4</td><td colspan="5">Checks breathing</td></tr>
<tr><td></td><td>Check for no breathing or agonal breathing</td><td colspan="2">2</td><td colspan="2">0</td></tr>
<tr><td></td><td>Confirm that the patient has not breathed for 5-10 seconds</td><td colspan="2">2</td><td colspan="2">0</td></tr>
<tr><td rowspan="2">5</td><td rowspan="2">If the patient has not breathed, begin CPR within 10 seconds</td><td>5~10s</td><td colspan="2">Over 10s</td><td>No CPR</td></tr>
<tr><td>4</td><td colspan="2">2</td><td>0</td></tr>
<tr><td>6</td><td colspan="5">CPR position</td></tr>
<tr><td></td><td>Place the heel of your hand in the right place for chest compression: in the inter-nipple line</td><td colspan="2">2</td><td colspan="2">0</td></tr>
<tr><td>7</td><td colspan="5">CPR posture</td></tr>
<tr><td></td><td>Place your other hand on top of the first hand</td><td colspan="2">2</td><td colspan="2">0</td></tr>
<tr><td></td><td>Keep the CPR arm straight</td><td colspan="2">2</td><td colspan="2">0</td></tr>
<tr><td></td><td>Keep the CPR arm perpendicular to the body</td><td colspan="2">2</td><td colspan="2">0</td></tr>
<tr><td>8</td><td colspan="5">Allow the student to continue chest compressions</td></tr>
<tr><td colspan="6">The examiner says: “The AED arrives on scene.”</td></tr>
<tr><td>9</td><td>After arrival of the AED, turns on the power of AED</td><td colspan="2">2</td><td colspan="2">0</td></tr>
<tr><td>10</td><td>Attaches the pads</td><td colspan="2">2</td><td colspan="2">0</td></tr>
<tr><td>11</td><td>Confirm that the pads are placed in the correct place</td><td colspan="2">4</td><td colspan="2">0</td></tr>
<tr><td>12</td><td>Before pushing the “shock” button, shout “keep clear”</td><td colspan="2">2</td><td colspan="2">0</td></tr>
<tr><td rowspan="2">13</td><td rowspan="2">After the protocol for AED, shock or analysis has been completed, resumes CPR immediately</td><td><5~10s</td><td colspan="2">Delay</td><td>No CPR</td></tr>
<tr><td>4</td><td colspan="2">2</td><td>0</td></tr>
<tr><td colspan="6">End the Test</td></tr>
</table>

Score:

Examiner’s Signature:
